# Percentage-Method Improves Properties of Workers’ Sitting- and Walking-Time Questionnaire

**DOI:** 10.2188/jea.JE20150169

**Published:** 2016-08-05

**Authors:** Tomoaki Matsuo, Hiroyuki Sasai, Rina So, Kazunori Ohkawara

**Affiliations:** 1 Hazard Evaluation and Epidemiology Research Group, National Institute of Occupational Safety and Health, Japan, Kawasaki, Kanagawa, Japan; 1 労働安全衛生総合研究所 産業疫学研究グループ; 2 Research Center for Overwork-Related Disorders, National Institute of Occupational Safety and Health, Japan, Kawasaki, Kanagawa, Japan; 2 労働安全衛生総合研究所 過労死等調査研究センター; 3 Faculty of Medicine, University of Tsukuba, Tsukuba, Japan; 3 筑波大学医学医療系; 4 Japan Society for the Promotion of Science, Tokyo, Japan; 4 日本学術振興会; 5 Graduate School of Comprehensive Human Sciences, University of Tsukuba, Tsukuba, Japan; 5 筑波大学大学院人間総合科学研究科; 6 Faculty of Informatics and Engineering, University of Electro-Communications, Chofu, Tokyo, Japan; 6 電気通信大学情報理工学部

**Keywords:** reliability, sedentary behavior, sitting time, validity, 信頼性, 座位行動, 座位時間, 妥当性

## Abstract

**Background:**

Does asking for the percentage of time spent sitting during work (P-method) instead of asking for the absolute length of time spent sitting (T-method) improve properties of the workers’ sitting- and walking-time questionnaire (WSWQ)? The purpose of this study was to investigate whether questioning technique influences test-retest reliability and criterion validity of the WSWQ.

**Methods:**

Sixty-five Japanese workers completed each version of the WSWQ in random order. Both questionnaires assessed quantities of time spent sitting or walking (including standing) during work time, non-working time on a workday, and anytime on a non-workday. Participants wore the thigh-worn inclinometer (activPAL) as criterion measure. Intraclass correlation coefficients (ICC) and Spearman’s ρ were used for the analyses.

**Results:**

For all three domains, values of reliability and validity with the P-method tended to be higher than with the T-method: ICC values ranged from 0.48–0.85 for the T-method and from 0.71–0.85 for the P-method; Spearman’s ρ values ranged from 0.25–0.58 for the T-method and from 0.42–0.65 for the P-method. The validities with both methods on a workday (0.51–0.58 for the T-method and 0.56–0.65 for the P-method) were higher than validities on a non-workday (0.25–0.45 for the T-method and 0.42–0.60 for the P-method). In post-survey interviews, 48 participants (77%) chose the P-method as their preferred questioning style.

**Conclusions:**

The study revealed that the P-method WSWQ had better reliability, validity, and ease of answering than the T-method, suggesting that the P-method can improve properties of the WSWQ and consequently advance the quality of epidemiological surveys in this field.

## Abstract (ja)

**【背景】:**

労働者の座位・歩行時間を評価する質問紙（Worker’s Sitting- and Walking-time Questionnaire: WSWQ）の質問方法として、座位時間を直接問う方法（時間法）ではなく、就業時間全体に対する座位時間の割合を問う方法（割合法）を用いることは有用だろうか。本研究では、質問方法の違いがWSWQの再検査信頼性や基準関連妥当性に及ぼす影響を検討した。

**【方法】:**

日本に在住する65名の労働者を対象に2種類（時間法と割合法）のWSWQによる調査を行った（順序をランダム化）。本研究で用いたWSWQは、時間法、割合法ともに、勤務中、勤務日の余暇時間、休日それぞれの座位時間および歩行時間（立位時間含む）を調査するものである。WSWQの妥当性を検証するため、対象者には身体活動量計（activPAL）の装着を求めた。信頼性の評価に級内相関係数（intraclass correlation coefficients: ICC）を、妥当性の評価にスピアマン順位相関係数（*ρ*）を算出した。

**【結果】:**

3つの時間領域全てにおいて、信頼性と妥当性の評価値はいずれも時間法より割合法が高い傾向にあった。すなわち、時間法のICC値は0.48～0.85、割合法のICC値は0.71～0.85であり、時間法の*ρ*値は0.25～0.58、割合法の*ρ*値は0.42～0.65であった。一方、時間法でも割合法でも、勤務日の妥当性評価値（時間法0.51～0.58；割合法0.56～0.65）は、休日の評価値（時間法0.25～0.45；割合法0.42～0.60）より高かった。また、質問方法に関する調査では、48名（77％）が時間法より割合法が回答しやすいと答えた。

**【結論】:**

本研究により、信頼性、妥当性ともに時間法より割合法の評価値が高い傾向にあること、また、対象者の多くが時間法より割合法が答えやすいと感じたことが明らかとなった。WSWQに割合法を用いることで質問紙の質が高まり、その結果、疫学調査そのものの質を高められるかもしれない。

## INTRODUCTION

People living in developed countries spend large parts of their waking time in sedentary behavior,^1^ especially in the workplace, where time spent seated at a desk has increased as development has increased.^2^ From a health perspective, addressing physical activity in the workplace environment is crucial, as many adults spend a large part of their total daily life in their workplace. Some studies have shown that occupational sitting time was associated with a higher risk of obesity,^3^ diabetes mellitus,^4^ cardiovascular disease,^5^ cancer,^6^^,^^7^ and mortality.^8^ Other studies, however, have shown no association between occupational physical activity and the risk of these diseases.^9^^,^^10^ Furthermore, some studies^11^^,^^12^ found an increased risk of disease in active workers compared to sedentary workers. Thus, whether occupational sitting time increases health risks is still controversial. A systematic review^13^ indicated that adequate techniques for measuring sitting time are needed to explain the discrepancies in the findings of these association studies.

Gibbs et al^14^ suggested the use of objective measures of sitting time, such as accelerometers, because of lower measurement error with both small sample size experiments and large population surveys. However, subjective measures, such as questionnaires, remain useful because they are more cost-effective and present a lower participant burden,^15^ although the key limitation of a questionnaire is poor validity with recall bias. Therefore, improving the validity of questionnaires is fundamental to improving epidemiological study in this field.

The International Physical Activity Questionnaire (IPAQ) is the most popular physical activity questionnaire in the world that can assess time spent sitting. However, a study^16^ showed that the convergent validity (vs accelerometers) was not sufficient (Spearman’s ρ, approximately 0.30). In general, questionnaires assessing sitting time, such as the IPAQ, use a questioning technique that asks for absolute length of time (hours and minutes) spent sitting (T-method), such as “How much time do you spend sitting on a week day?” However, for the responders, recalling the hours and minutes of time spent sitting can be difficult and may be an important reason for the low validity of these questionnaires. Chau et al^17^ used the ActiGraph accelerometer (ActiGraph LLC, Pensacola, FL, USA) as a criterion measure and showed that the criterion validity of the percentage method (P-method; asking for the percentage of time spent sitting) was higher than that of the T-method.

Although the ActiGraph is widely used as a criterion measure for a physical activity questionnaire, the device does not adequately distinguish postures such as sitting and standing. Kozey-Keadle et al^18^ showed that the thigh-worn activPAL inclinometer (PALtechnologies, Glasgow, Scotland) more precisely assessed subjects’ sedentary behavior compared with the hip-mounted ActiGraph accelerometer. Furthermore, Grant et al^19^ showed a mean percentage difference of 0.19% in sitting time between the activPAL and direct observation. Thus, the thigh-worn inclinometer may be the best criterion device currently available for testing validity of a sitting time questionnaire.^15^

The purpose of this study was to compare the reliability and validity of the T-method and P-method questioning techniques for the workers’ sitting- and walking-time questionnaire (WSWQ) using the activPAL inclinometer as a criterion device. We tested the hypothesis that P-method would have a higher validity than T-method because it would be easier for participants to respond accurately to the P-method compared with the T-method.

## METHODS

### Participant recruitment

We recruited participants through the local newspaper and by word-of-mouth in the area surrounding the University of Tsukuba, Ibaraki, Japan. The inclusion criteria in this study were 1) Japanese-language proficiency, 2) aged 20–60 years old, and 3) employed in part-time or full-time work at least 3 days a week. We made sure to include several job categories when recruiting participants. Sixty-five Japanese workers participated in this study and completed all of the study protocol. This study was conducted in accordance with the guidelines proposed in the Declaration of Helsinki. The Ethics Committee of the National Institute of Occupational Safety and Health, Japan reviewed and approved the study protocol (ID H2523). The aim and design of this study were explained to every participant before each gave their written informed consent. In return for participation, participants received a payment of ¥10 000 (Japanese yen). Table 1 shows participant characteristics.

**Table 1.** Descriptive characteristics of the study participants

	Men (*n* = 36)	Women (*n* = 26)
Age, years	46.3 (8.0)	35.8 (7.5)
Body mass index, kg/m^2^	25.0 (2.6)	21.0 (2.7)
Education, post-high school, *n* (%)	31 (86.1)	20 (76.9)
Married, *n* (%)	33 (91.7)	17 (65.4)
Workdays^a^	4.5 (0.8)	4.2 (1.0)
Non-workdays (days-off)^a^	1.4 (0.7)	2.4 (0.8)
Worktime^b^ per day indicated by log, min	661 (101)	602 (107)
Participants’ occupations, *n*		
Clerical job	6	16
Civil-service worker	3	0
Construction service	1	0
Driver	1	0
Engineer	6	0
Hotel service	2	0
Management level employee	2	0
Nurse	0	3
Physical therapist/Physical educator	1	6
Researcher	7	1
Sales and marketing	5	0
Teacher	2	0

Values are presented as *n* (%) or mean (standard deviation).

^a^Valid workdays and non-workdays were calculated using each participant’s daily log information.

^b^From time arriving at work place to time leaving work place.

### Data collection

Participants completed both versions of the WSWQ on the first day (time 1) and again one week later (time 2). For time 1, half of the participants were randomly selected to start with the T-method followed by the P-method, while the other half started with the P-method followed by the T-method. For time 2, participants completed the two versions of the questionnaire in the same order as they had previously. Participants wore the activPAL monitor 24 hours per day for the 7 days between the time 1 and time 2 questionnaire assessments.

### Measures

#### Questionnaire

The WSWQ is a self-administered questionnaire that can measure time spent sitting and walking (including standing) separately in three different domains covering a worker’s typical weekly life: (a) working time; (b) non-working time, such as leisure time, on a workday; and (c) non-workday time. The WSWQ also includes questions about participants’ age, gender, height, weight, education level, marital status, weekly exercise habits, commuting means and time, and job title. The T-method WSWQ directly asks for length of time (hours and minutes) spent sitting and walking/standing on a typical day in the previous month: “How much time do you spend sitting on a typical day during your working hours?” (see eAppendix 1). The P-method WSWQ asks the participant for the proportion of time spent sitting or walking/standing in a particular time period (eg, total work time per day): “What proportion of a typical day do you spend sitting during your working hours?” The P-method WSWQ also asks for bedtime, rising time, work start time, and work end time on a typical day in the previous month (see eAppendix 2). Once we learn the proportional time a participant spends sitting or walking/standing, we can calculate the number of minutes per day participants spent sitting or walking/standing for each of the three domains. The proportion of each activity (sitting and walking/standing) was multiplied by the total minutes of each domain (working time, non-working time on a workday, non-workday time). For example, *“sitting time during working time” = total working time (min) × reported proportion of sitting time (%)*; *“sitting time during non-work time on a workday” = {1440 min (ie, 24 h) − sleeping time (min) − working time (min) − commuting time (min)} × reported proportion of sitting time (%)*; *“sitting time on non-workday” = {1440 min − sleeping time (min)} × reported proportion of sitting time (%)*.

In post-survey interviews, the participants reported their preference of questioning technique by answering the question: “When responding to the survey questions, did you prefer answering in fixed lengths (hours and minutes) of time or as percentages (%) of time?”

#### Criterion measure (activPAL)

The activPAL^3 ^™ (PAL Technologies Ltd, Glasgow, Scotland) is a small, light inclinometer that continuously records subjects’ posture, such as sitting/lying, standing, or stepping. We waterproofed the device using a nitrile sleeve and cling film in accordance with the manufacturer’s instructions. Participants attached the waterproofed activPAL directly on their skin at mid-thigh using 3M Tegaderm™ tape. We requested they wear the device 24 hours a day over a 7-day period. Participants received an instruction leaflet and 3M Tegaderm™ tape so they could adjust and reattach the device if it was uncomfortable or irritating. During the 7-day measurement period, participants were also instructed to record a daily log of particular times during the day, such as bedtime and rising time, workday or non-workday, work start and end times, normal or unusual workday, and any periods they may not have worn the activPAL.

The activPAL data can be exported into a Microsoft Excel file using the activPAL software (version 7.2.32). The software provided us with detailed time data (15-s intervals from 0:00 to 24:00 h) on each measurement day. We calculated each subject’s average time spent sitting/lying, standing, and stepping using both activPAL data and the daily log information. If we found a day recorded as an unusual working day, such as business trip or a half day off, or if the participants failed to record a needed time, the day’s data were removed from the average daily calculation.

### Data analysis

For the analyses, we excluded three participants because of technical problems with the activPAL or insufficient valid criterion data (at least 3 valid workdays). Consequently, 62 participants were included in the final analyses.

The one week interval test-retest reliability of the questionnaires was examined using intraclass correlation coefficients (ICC) and 95% confidence intervals (CIs), with an ICC <0.40 indicating poor repeatability, 0.40–0.75 indicating fair to good repeatability and >0.75 indicating excellent repeatability.^20^ We examined the criterion validity (Spearman’s ρ) of the questionnaires by comparing the values from the questionnaires at both time 1 and time 2 with the values from activPAL. The ρ values were interpreted as follows: <0.30 indicated weak, 0.30–0.49 indicated low, 0.50–0.69 indicated moderate, 0.70–0.89 indicated strong, and ≥0.90 indicated very strong correlation.^21^ We used Bland-Altman plots to visually assess bias.^22^ Participants were classified into four groups using quartile points, and we calculated the Cicchetti-Allison’s weighted kappa coefficient to assess degree of agreement between the questionnaire and activPAL classifications.

Values are expressed as *n* (%), median (25%–75%), or mean (standard deviation), as appropriate. For the analyses, *P*-value <0.05 was considered statistically significant. We used SAS, version 9.3 (SAS Institute Japan, Tokyo, Japan) to analyze the data.

## RESULTS

Table 1 shows the demographic characteristics of the participants. Most participants reported that they were married with post-high school education. We observed slightly higher age and body mass index in male participants than in female participants. While most female participants worked in clerical jobs, male participants worked in various types of jobs.

Table 2 shows the test-retest reliabilities of both the T-method and P-method questionnaires. ICC values ranged from 0.48–0.85 for the T-method and from 0.71–0.85 for the P-method. During working time, both the T-method and the P-method had excellent ICCs for both sitting time and walking/standing time. During non-working time on a workday, the ICCs in the P-method were relatively higher than in the T-method for both sitting time and for walking/standing time: the ICCs for the P-method were fair to good (sitting time) or excellent (walking/standing), whereas those for the T-method were fair to good. There was a similar trend on non-workdays: the ICCs for the P-method were excellent, whereas those for the T-method were fair to good. The lowest reliability (0.48) was in the T-method for walking/standing time on non-workdays.

**Table 2.** Test-retest reliability of values measured by each questionnaire method at times 1 and 2

		Time 1		Time 2		ICC	95% CI
	
		Median	IQR^a^	Median	IQR		
		(minutes per day)		(minutes per day)			
Workday							
During working time							
Sitting	Time method	428	360–510	420	330–490	0.85	0.76–0.91
	Percentage method	496	404–574	470	360–540	0.83	0.73–0.89
	activPAL	412	352–502				
Walking/Standing	Time method	105	45–240	120	60–180	0.83	0.73–0.89
	Percentage method	101	61–257	144	71–247	0.85	0.76–0.90
	activPAL	186	135–281				
During non-working time^a^							
Sitting	Time method	200	120–240	180	120–240	0.49	0.28–0.66
	Percentage method	202	132–284	215	132–264	0.71	0.56–0.81
	activPAL	184	137–230				
Walking/Standing	Time method	60	30–120	60	60–210	0.56	0.37–0.71
	Percentage method	81	38–174	80	33–172	0.77	0.65–0.85
	activPAL	107	76–183				

Non-workday^b^							
Sitting	Time method	480	300–660	600	300–660	0.64	0.47–0.76
	Percentage method	576	408–683	603	315–714	0.78	0.66–0.86
	activPAL	590	495–677				
Walking/Standing	Time method	300	240–540	360	180–420	0.48	0.27–0.65
	Percentage method	384	288–576	390	222–648	0.79	0.68–0.87
	activPAL	370	294–434				

CI, confidence interval; ICC, intraclass correlation coefficient; IQR, interquartile range.

^a^Excluding working, commuting and sleeping time.

^b^Excluding sleeping time.

Table 3 shows the Spearman’s ρ as a validity value comparing criterion activPAL data and questionnaire responses at both time 1 (before the activPAL measurement) and time 2 (after the activPAL measurement). Table 3 also shows kappa coefficients indicating the degree of agreement between questionnaire and activPAL classifications.

**Table 3.** Criterion validity of values measured by each questionnaire method compared with values measured by the activPAL

	Time 1^a^				Time 2^b^			
	
	Spearman’s ρ		Kappa coefficient^c^		Spearman’s ρ		Kappa coefficient^c^	
			
	T-method	P-method	T-method	P-method	T-method	P-method	T-method	P-method
Workday								
During working time								
Sitting	0.52*	0.59*	0.39*	0.44*	0.56*	0.65*	0.41*	0.44*
Walking/Standing	0.56*	0.56*	0.38*	0.35*	0.58*	0.60*	0.40*	0.45*
During non-working time^d^								
Sitting	0.55*	0.57*	0.43*	0.36*	0.51*	0.60*	0.36*	0.41*
Walking/Standing	0.58*	0.61*	0.35*	0.40*	0.57*	0.61*	0.39*	0.45*
Non-workday^e^								
Sitting	0.25	0.42*	0.13	0.23*	0.37*	0.53*	0.28*	0.32*
Walking/Standing	0.30*	0.45*	0.16*	0.21*	0.45*	0.60*	0.31*	0.34*

T-method, time method (questionnaire); P-method, percentage method (questionnaire).

^a^Time 1: Subjects completed the questionnaire before the activPAL measurements.

^b^Time 2: Subjects completed the questionnaire after the activPAL measurements.

^c^Participants were classified into four groups using quartile points.

^d^Excluding working, commuting, and sleeping time.

^e^Excluding sleeping time.

*P < 0.05.

Spearman’s ρ values ranged from 0.25–0.58 for the T-method and from 0.42–0.65 for the P-method. On workdays, both the T-method and P-method had moderate validities, while the ρ values for the P-method were relatively higher than the ρ values for the T-method. On non-workdays, we observed weak or low validities for the T-method, whereas we observed low or moderate validities for the P-method. The highest ρ value (0.65) was for the P-method for sitting time during work in the time 2 questionnaire, and the lowest ρ value (0.25) was for the T-method for sitting time on non-workdays in the time 1 questionnaire. There was a similar trend with kappa coefficient values. The values ranged from 0.13–0.43 for the T-method and from 0.21–0.45 for the P-method. The values of all three domains using the P-method were higher than using the T-method, except for walking/standing time at work and sitting time during non-working time on a workday for the time 1 questionnaire.

The validities (ρ values and kappa coefficient values) on workdays with both T-method and P-method were higher than those on non-workdays. The validity values for the time 2 questionnaire tended to be higher than for the time 1 questionnaire.

**Figure 1.**
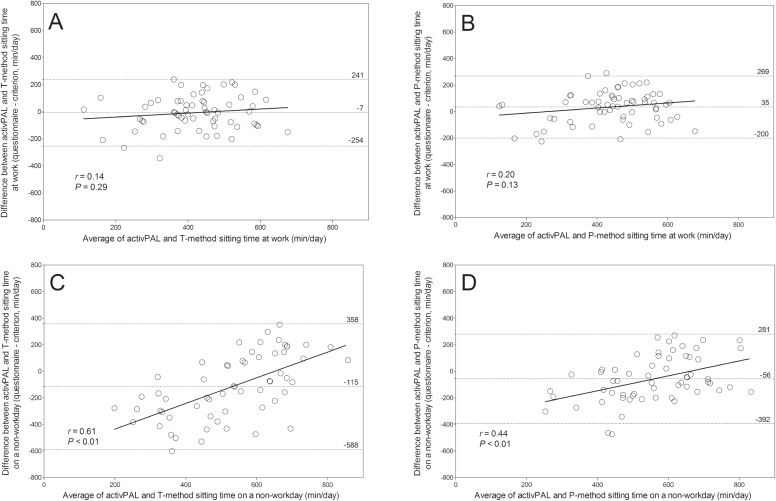
Bland-Altman plot comparing time 1 questionnaire sitting time with the criterion sitting time (activPAL with daily log). (A) T-method sitting time at work; (B) P-method sitting time at work; (C) T-method sitting time on a non-workday; (D) P-method sitting time on a non-workday. The mean difference and the 95% limits of agreement appear as dashed lines. Regression line and correlation coefficients between X and Y are displayed. CI, confidence interval; P-method, percentage method (questionnaire); T-method, time method (questionnaire).

Figure 1 shows the Bland-Altman plots comparing time 1 questionnaire-recorded sitting time and activPAL sitting time. The mean differences between questionnaire sitting time at work and activPAL sitting time at work for the T-method and P-method were −6.7 min/day (*P* = 0.68) (Figure 1A) and 34.5 min/day (*P* = 0.03) (Figure 1B), respectively. For work time, there were similar patterns between the T-method (Figure 1A) and P-method (Figure 1B) plots, although the fixed bias (ie, overestimation) with the P-method was significant. The mean differences between questionnaire sitting time during a non-workday and activPAL sitting time during a non-workday for the T-method and P-method were −114.7 min/day (*P* < 0.01) (Figure 1C) and −55.7 min/day (*P* = 0.02) (Figure 1D), respectively. On non-workdays, there were significant fixed (ie, underestimation) and proportional biases (biases increased at higher levels of sitting time) in both plots, but the biases with the T-method (Figure 1C) were larger than with the P-method (Figure 1D).

In the post-experiment interview regarding the questioning technique, 48 participants (77%) chose P-method as their preferred questioning style.

## DISCUSSION

The purpose of this study was to investigate whether questioning technique would influence the measurement properties of questionnaires measuring workers’ sitting and walking/standing times in three domains of their life (working time, non-working time on a workday, and during a non-working day) using thigh-worn activPALs as a criterion measure. Results suggest that the P-method had relatively better measurement properties than the T-method. In addition, the study revealed that 77% of the participants preferred the P-method compared with the T-method when responding to the questions. Ease of answering questions is an important factor to consider for improving the quality of epidemiological studies. Our study suggests that the ease of answering questions may have an influence on the difference between questionnaire properties in the T-method and P-method. The study also suggests that the validities on a non-workday were substantially lower than validities on a workday, which was especially pronounced in the T-method. Overall, the present study suggests that the P-method would be a better questioning technique than the T-method for measuring workers’ sitting and walking times on workdays and non-workdays.

Chau et al^17^ developed the Occupational Sitting and Physical Activity Questionnaire (OSPAQ), taking their cue from workplace ergonomics studies.^23^^,^^24^ The OSPAQ asked participants to estimate the percentage of time spent sitting, standing, and engaged in physical activity at work, similar to the P-method in our study. Their study^17^ showed that the OSPAQ had better measurement properties (reliabilities of 0.73–0.97 and validities of 0.29–0.65) than the other type of questionnaire (reliabilities of 0.54–0.89 and validities of 0.27–0.52), which asked for the actual length of time spent sitting, standing, and engaged in physical activity at work, similar to the T-method in our study. The results of Chau et al^17^ are consistent with our study. However, their study used the ActiGraph accelerometer as the criterion device rather than the activPAL inclinometer. Moreover, the participants of their study wore the ActiGraph for 7 days between the first and second questionnaire assessments, and the time 2 questionnaire responses (ie, after 1-week ActiGraph measurements) were used for the validity analyses. Although this research method was logical because the OSPAQ asked for the worker’s sitting time “in the last 7 days”, it is possible that their validity values may have been overestimated. Our present study showed that the validity values at the time 2 questionnaire tended to be higher than those at the time 1 questionnaire, suggesting that the one week of measuring with the criterion device and recording a daily log can affect the validity values for the subsequent questionnaire.

A recent study by Chastin et al^25^ used the activPAL as the criterion device and assessed measurement properties of IPAQ’s sitting items (ie, the T-method). They showed remarkably low correlations (0.11–0.28) between the IPAQ and the activPAL. The weak validities might have occurred because the study only measured overall rather than domain-specific sitting time on weekdays and weekend days. Furthermore, participants in their study did not maintain a daily log. Sleeping time, for example, was estimated using activPAL data showing a long continuous period of non-upright posture. These methodological limitations may have caused the low validities because using a daily log with activPAL is highly recommended.^26^^,^^27^

In our study, we saw higher validities during workdays compared to non-workdays with both the T-method and P-method (Table 3), and biases between questionnaire-recorded sitting time and activPAL sitting time at work (Figure 1A and Figure 1B) were smaller than biases on non-work days (Figure 1C and Figure 1D). For many participants, recalling the time they spend sitting at work may be easier because work activity is often routine compared to unstructured activities, such as on their days off. This is consistent with other studies.^27^^,^^28^ Marshall et al^28^ found that validities of their sitting time questionnaire were higher for weekdays than for weekend days. Healy et al^27^ also indicated that validities of measurements of sedentary time tended to be higher for domain-specific measures than for overall measures. The lowest ρ value (0.25) in our study was for sitting time on a non-workday using the T-method. Difficulty in recalling an absolute length of time spent sitting (T-method) without a domain-specific measure for a non-workday may be the primary reason for the low validity.

In their systematic review article of occupational sitting and health risks, van Uffelen et al^13^ indicated that remarkably few studies reported on the reliability and validity of their sitting time measures, which generated inconsistent results across the existing epidemiological studies. They also indicated that many studies used a categorical measure of occupational activity, which made it difficult to perform dose-response analyses, and that sitting time as a continuous variable should be considered even when using questionnaires. On this point, the P-method in our study had acceptable reliabilities and validities and could quantify time spent sitting. Although objective measures, such as accelerometers, are recommended to precisely measure sitting time, subjective measurements, such as questionnaires, still have advantages in cost and subject burden for large population surveys. Therefore, we believe our study results add valuable information for future research in this field.

The primary strength of this study is that we used the thigh-worn activPAL inclinometer as the criterion device rather than a hip-mounted accelerometer, such as ActiGraph; some studies^18^^,^^29^ indicate that the activPAL is a better device to assess sitting time compared with the ActiGraph. On the other hand, there are some limitations of this study. First, participants were not enrolled as a random sample from the general population but as a convenience sample; as such, the range of participants’ job categories was limited, and male participants’ BMIs were relatively high, which may have resulted in biases. Second, participant response bias may have occurred because the participants answered both types of questionnaire in randomly assigned order (T-method followed by P-method or P-method followed by T-method) on both assessment days. Answers the participants gave in the first test on the assessment day might influence the responses they gave in the second test. However, we believe the random order technique used in this study should attenuate this potential bias. Third, some participants had no days off or only one non-workday during the 7-day measuring period. Therefore, the average number of non-workdays for men and women were only 1.4 and 2.4 days, respectively (Table 1), which may make it difficult to generalize non-workday activities.

In conclusion, the better validity seen with the P-method compared to the T-method supports our hypothesis that it would be easier for participants to respond accurately to the P-method compared with the T-method. The difficulty in recalling an absolute length of time spent sitting compared to a percentage of time is a potential reason for this difference. Using the P-method improved properties of the WSWQ, and most participants preferred the P-method rather than the T-method questionnaire. This suggests that using the P-method may improve the quality of epidemiological surveys that investigate the association between workers’ physical activity and health.

## ONLINE ONLY MATERIALS

eAppendix 1. Time method.

eAppendix 2. Percentage method.

Abstract in Japanese.
