# Supplementary material for: Percentage-Method Improves Properties of Workers’ Sitting- and Walking-Time Questionnaire
Source: J Epidemiol. 2016 Aug 5;26(8):405–12. doi: 10.2188/jea.JE20150169 (PMC4967661; doi:10.2188/jea.JE20150169)
Supplement: eAppendix 1. [file je-26-405-s001.pdf]

eAppendix 1. Time method

*This is an English translated version. The Japanese version was used in the study.

The following questions pertain to **workdays**. Thinking back on the last month or so, imagine your **typical workday** and answer the following questions.

Q1 How long do you spend **sitting** and **standing or walking** on a typical day **<u>during your working hours</u>** (excluding time spent commuting)?

A) Time spent sitting ( ) h ( ) min
B) Time spent standing or walking ( ) h ( ) min

Q2 How much of **<u>your free time—not spent sleeping, commuting, or working—on workdays</u>** (such as after work, when doing housework, or at home) do you spend **sitting or reclining** and **standing or walking**?

A) Time spent sitting or reclining ( ) h ( ) min
B) Time spent standing or walking ( ) h ( ) min

The following question pertains to **non-workdays** (days off from work). Thinking back on the last month or so, imagine your **typical non-workday** and answer the following question.

Q3 How much of **<u>your time—not spent sleeping—on a non-workday</u>** (including housework and gardening) do you spend **sitting or reclining** and **standing or walking**?

A) Time spent sitting or reclining ( ) h ( ) min
B) Time spent standing or walking ( ) h ( ) min
