# Supplementary material for: Percentage-Method Improves Properties of Workers’ Sitting- and Walking-Time Questionnaire
Source: J Epidemiol. 2016 Aug 5;26(8):405–12. doi: 10.2188/jea.JE20150169 (PMC4967661; doi:10.2188/jea.JE20150169)
Supplement: eAppendix 2. [file je-26-405-s002.pdf]

eAppendix 2. Percentage method

*This is an English translated version. The Japanese version was used in the study.

The following questions pertain to **workdays**. Thinking back on the last month or so, imagine **your typical workday** and answer the following questions.

Q1 What time do **you go to bed** on **the day before a workday**? (Getting in bed after midnight should be considered as part of the previous day.) Consider this as the time you get into bed (when you lie down under the bedcovers), not the time you fall asleep. Also, what time do **you rise** **on workdays**? Consider this as the time you get out of bed, not the time you wake up.

Bedtime on the day before a workday (:)
Rising time on a workday (:)

Q2 What time do **you leave the house** for work **on workdays**? What time do **you arrive at work**? Check (✓) next to "I do not commute," and write the time you start work if you do not commute. This may apply if you are a homemaker or if you work from home.

Time leaving the house (:)
Time arriving at work (:)
I do not commute ( ) ⇨ Time to start work (:)

Q3 What time do **you leave your workplace after completing your work on a typical workday? (:)**

Q4 What proportion of a typical day do you spend **sitting** and **standing or walking** **during your working hours** (excluding time spent commuting)? Consider 100% as the entire time spent working and respond so that both fields total 100%.

A) Time spent sitting ( ) %
B) Time spent standing or walking ( ) %

Q5 What proportion of **your free time—not spent sleeping, commuting, or working—on workdays** (such as after work, when doing housework, or at home) do you spend **sitting or reclining** and **standing or walking**? Consider 100% as the total amount of free time on workdays and respond so that both fields total 100%.

A) Time spent sitting or reclining ( ) %
B) Time spent standing or walking ( ) %

The following questions pertain to **non-workdays** (days off from work). Thinking back on the last month or so, imagine your **typical non-workday** and answer the following questions.

Q6 What **time do you go to bed** **on the day before a non-workday**? (Getting in bed after midnight should be considered as part of the previous day.) Consider this as the time you get into bed (when you lie down under the bedcovers), not the time you fall asleep. Also, what time do **you rise** on a **non-workday**? Consider this as the time you get out of bed, not the time you wake up.

Bedtime the day before a non-workday (:)
Rising time on a non-workday (:)

Q7 What proportion of **your time—not spent sleeping—on a non-workday** (including housework and gardening) do you spend **sitting or reclining** and **standing or walking**? Respond so that both fields total 100%.

A) Time spent sitting or reclining ( ) %
B) Time spent standing or walking ( ) %
