# Supplementary material for: Percentage-Method Improves Properties of Workers’ Sitting- and Walking-Time Questionnaire
Source: J Epidemiol. 2016 Aug 5;26(8):405–12. doi: 10.2188/jea.JE20150169 (PMC4967661; doi:10.2188/jea.JE20150169)
Supplement: Abstract in Japanese. [file je-26-405-s003.pdf]

労働者の座位・歩行時間を評価する質問紙に割合法を用いた場合の有用性について

松尾知明 [1,2], 笹井浩行 [3, 4], 蘇リナ [2,4,5], 大河原一憲 [6]

[1] 労働安全衛生総合研究所 産業疫学研究グループ； [2] 労働安全衛生総合研究所 過労死等調査研究センター； [3] 筑波大学医学医療系； [4] 日本学術振興会； [5] 筑波大学大学院人間総合科学研究科； [6] 電気通信大学情報理工学部

【背景】労働者の座位・歩行時間を評価する質問紙（Worker’s Sitting- and Walking-time Questionnaire: WSWQ）の質問方法として、座位時間を直接問う方法（時間法）ではなく、就業時間全体に対する座位時間の割合を問う方法（割合法）を用いることは有用だろうか。本研究では、質問方法の違いが WSWQ の再検査信頼性や基準関連妥当性に及ぼす影響を検討した。

【方法】日本に在住する 65 名の労働者を対象に 2 種類（時間法と割合法）の WSWQ による調査を行った（順序をランダム化）。本研究で用いた WSWQ は、時間法、割合法ともに、勤務中、勤務日の余暇時間、休日それぞれの座位時間および歩行時間（立位時間含む）を調査するものである。WSWQ の妥当性を検証するため、対象者には身体活動量計（activPAL）の装着を求めた。信頼性の評価に級内相関係数（intraclass correlation coefficients: ICC）を、妥当性の評価にスピアマン順位相関係数（ρ）を算出した。

【結果】3 つの時間領域全てにおいて、信頼性と妥当性の評価値はいずれも時間法より割合法が高い傾向にあった。すなわち、時間法の ICC 値は 0.48～0.85、割合法の ICC 値は 0.71～0.85 であり、時間法の ρ 値は 0.25～0.58、割合法の ρ 値は 0.42～0.65 であった。一方、時間法でも割合法でも、勤務日の妥当性評価値（時間法 0.51～0.58；割合法 0.56～0.65）は、休日の評価値（時間法 0.25～0.45；割合法 0.42～0.60）より高かった。また、質問方法に関する調査では、48 名（77%）が時間法より割合法が回答しやすいと答えた。

【結論】本研究により、信頼性、妥当性ともに時間法より割合法の評価値が高い傾向にあること、また、対象者の多くが時間法より割合法が答えやすいと感じたことが明らかとなった。WSWQ に割合法を用いることで質問紙の質が高まり、その結果、疫学調査そのものの質を高められるかもしれない。

キーワード：信頼性；座位行動；座位時間；妥当性
